# Role of mitochondria-associated membranes (MAMs) in inflammatory signaling: implications for acute lung injury pathogenesis

**DOI:** 10.3389/fcell.2025.1690934

**Published:** 2025-11-24

**Authors:** Huifeng Qian, Guanguan Qiu, Jianguo Xu, Guoping Zheng

**Affiliations:** Shaoxing Second Hospital, Shaoxing, Zhejiang, China

**Keywords:** mitochondria-associated membranes (MAMs), mitochondria, endoplasmic reticulum, inflammation, acute lung injury

## Abstract

Mitochondria-associated membranes (MAMs), the specialized contact regions linking the endoplasmic reticulum (ER) and mitochondria, have emerged as dynamic communication hubs critical for preserving cellular homeostasis. These structures are crucial for controlling a range of essential cellular processes, such as calcium (Ca^2+^) signaling, lipid metabolism, autophagy, apoptosis, and inflammatory response. Increasing evidence connects MAM dysfunction to various inflammatory conditions, such as metabolic disorders, neurodegenerative diseases, and antiviral response. In the context of acute lung injury, altered ER–mitochondria interactions can result in mitochondria Ca^2+^ overload, heightened oxidative stress, and augmented inflammatory response. Together, these pathological processes enhance endothelial permeability and exacerbate pulmonary inflammation. This review highlights the structural and functional attributes of MAMs, the mechanisms underlying MAM-mediated inflammation, and the roles of MAMs in metabolic, neurological, and antiviral inflammation. It also delves into the role of MAMs in acute lung injury, unveiling fresh insights that may pave the way for innovative therapies targeting ER–mitochondria crosstalk.

## 1 Introduction

Acute lung injury (ALI) and its most severe progression, acute respiratory distress syndrome (ARDS), are main contributors to respiratory failure in intensive care units. Despite the implementation of supportive strategies such as lung-protective mechanical ventilation, prone positioning, fluid management, optimized PEEP settings, neuromuscular blockade, and extracorporeal membrane oxygenation (ECMO), the mortality rate for ARDS continues to stay around 30%–40% globally (McNicholas et al., 2018). Between 2020 and 2023, ARDS was a leading cause of death among COVID-19 patients, with a range of mortality rate from 54% to 76% (Aweimer et al., 2023). In the early stages of lung injury, alveolar macrophages are activated in response to various pathogens and tissue damage through pattern recognition receptors. This activation prompts the secretion of proinflammatory cytokines, which in turn stimulate surrounding alveolar epithelial cells and tissue macrophages to release chemokines. Subsequently, excessive neutrophils and macrophages are recruited to the inflammation site, exacerbating the cytokine storm. Ultimately, the inflammatory response compromises the barrier function of the endothelium and alveolar epithelium, leading to fluid accumulation in the airspaces (Matthay et al., 2019).

During the past several decades, research has shown that approximately 5%–20% of the outer mitochondrial membrane (OMM) is physically linked to the endoplasmic reticulum (ER), resulting in the formation of specialized contact regions known as mitochondria-associated membranes (MAMs) (Barazzuol et al., 2021). These contacts can be isolated by subcellular fractionation and visualized via techniques such as transmission electron microscopy (Thi et al., 2023). MAMs, roughly 10–30 nm apart, form a communication bridge between ER and mitochondria. They play a vital function in various cellular functions such as calcium (Ca^2+^) homeostasis, inflammasome activation, ER stress, mitochondria dynamics, apoptosis, and lipid metabolism (van Vliet et al., 2014). There are more than 1,000 proteins present in the MAMs by mass spectrometry analysis (Sala-Vila et al., 2016). Malfunction of MAMs has been associated with the development of a variety of inflammation-associated diseases such as diabetes, non-alcoholic fatty liver disease (NAFLD), Alzheimer’s disease, and Parkinsonian syndromes (Jiang et al., 2023).

Accumulating evidence links alterations in MAMs to inflammatory response and the pathophysiology of acute lung injury. Dysregulated ER–mitochondria communication at MAMs has been shown to result in mitochondrial Ca^2+^ accumulation, oxidative stress, and proinflammatory signaling. In the present review, we seek to provide an updated overview of MAMs in the context of inflammation and acute lung injury, highlighting their potential as novel targets for treating acute lung injury.

## 2 Structure and composition of MAMs

**FIGURE 1:**
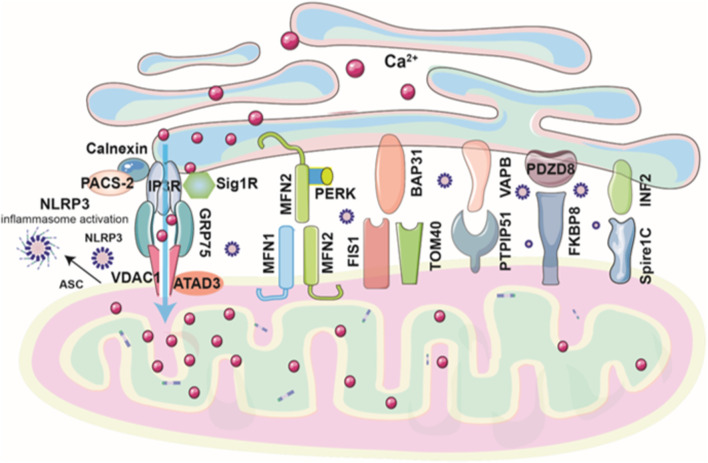
Schematic diagram of ER–mitochondria tethering complexes at MAMs. ER–mitochondria tethering at MAMs is mediated primarily by six key protein complexes that regulate Ca^2+^ signaling, lipid exchange, mitochondrial dynamics, and cellular stress responses. The IP3R–GRP75–VDAC1 complex forms the core Ca^2+^ transfer axis, modulated by modulators such as Sig1R, calnexin, PACS-2, ATAD3, and others. The mitofusin complex (Mfn2–Mfn1/Mfn2) tethers ER and mitochondria and interacts with PERK to regulate stress signaling. The BAP31–Fis1/TOM40 complex links apoptosis and mitochondrial respiration. VAPB–PTPIP51 coordinates Ca^2+^ transfer and autophagy, disrupted in neurodegenerative disease. PDZD8–FKBP8 supports ER–mitochondria apposition and neuronal Ca^2+^ signaling, while the INF2–Spire1C complex promotes actin-driven mitochondrial fission.

Many tethering or bridge proteins facilitate the interaction between the ER and mitochondria at MAMs (Figure 1). These proteins are essential for regulating critical cellular functions including lipid exchange, reactive oxygen species (ROS) regulation, Ca^2+^ transport, and mitochondrial dynamics (Table 1). First, the complex of inositol 1,4,5-trisphosphate receptor (IP3R), voltage-dependent anion channel 1 (VDAC1), and the chaperone GRP75 plays a role in regulating the ER-mitochondria apposition (Barazzuol et al., 2021). The IP3R mediates the release of Ca^2+^ from the ER (Berridge, 2016), while VDAC1 functions as a channel for mitochondrial Ca^2+^ uptake at the OMM (Conti et al., 2024). Ca^2+^ ions are efficiently transferred across the ER-mitochondria interface through the IP3R-GRP75-VDAC1 axis, utilizing the mitochondrial Ca^2+^ uniporter (MCU), a highly selective channel embedded in the inner mitochondrial membrane. Silencing GRP75 blocked Ca^2+^ accumulation in mitochondria, underscoring the critical role of GRP75 in mediating conformational coupling between IP3R and VDAC1 (Szabadkai et al., 2006). Under normal conditions, the ER protein sigma-1 receptor (Sig1R) binds with GRP78 (BiP) and serves as a Ca^2+^-sensitive chaperone at MAMs. During ER stress, Sig1R dissociates from GRP78 and associates with IP3R, thereby enhancing ER-mitochondrial Ca^2+^ signaling and cell survival (Hayashi and Su, 2007). Calnexin, a molecular chaperone in the ER, interacts with IP3R to coordinate Ca^2+^ signaling to the mitochondria (Joseph et al., 1999). Phosphofurin acidic cluster sorting protein 2 (PACS-2) interacts with calnexin and regulates its localization across the ER, MAMs, and the plasma membrane, ensuring proper distribution and function (Myhill et al., 2008). Meanwhile, ATAD3, a mitochondrial protein, contributes to MAM formation that facilitates ER-mitochondria cholesterol transfer, crucial for steroidogenesis in Leydig cells (Issop et al., 2015). ATAD3 also binds with IP3R1-GRP75-VDAC1 complex, inhibiting MAMs-mediated mitochondrial Ca^2+^ overload and mitochondrial dysfunction, which helps alleviate isoproterenol-induced cardiac hypertrophy (Li Z. et al., 2024). The second ER-mitochondria bridge is established through the interaction of mitofusin 2 (Mfn2), a GTPase located on the ER membrane, with Mfn1 or Mfn2 present on the OMM. Silencing of Mfn2 weakens ER-mitochondria association and impairs mitochondrial Ca^2+^ uptake after IP3 stimulation (de Brito and Scorrano, 2008). Mfn2 physically interacts with protein kinase RNA-like ER kinase (PERK), an ER stress sensor, to maintain the inactive state of PERK. Downregulation of Mfn2 leads to PERK activation, which in turn triggers the unfolded protein response to restore homeostasis in the ER and mitochondria (Munoz et al., 2013). Deletion of PERK results in abnormal ER morphology, diminished Ca^2+^ signaling, and weakened ER-mitochondria tethering. Additionally, the absence of PERK alleviates apoptosis triggered by ROS-mediated ER stress (Verfaillie et al., 2012). In the third ER-mitochondria tethering complex, the B-cell receptor-associated protein 31 (BAP31), situated on the ER membrane, promotes ER-mitochondria communication by binding with fission protein 1 (Fis 1) and TOM40 on the OMM (Iwasawa et al., 2011; Namba, 2019). The FIS1-BAP31 interaction induces the cleavage of BAP31, recruitment of procaspase‐8, and Ca^2+^ release from ER-mitochondria (Iwasawa et al., 2011). The BAP31-Tom40 interaction enhances the function of mitochondrial complex I activity and oxygen consumption by facilitating the translocation of NDUFS4 to the mitochondria (Namba, 2019). A fourth ER-mitochondria tethering mechanism involves the ER protein VAPB and the mitochondrial protein PTPIP51, which regulate autophagy and Ca^2+^ transfer through IP3R-mediated pathways (De Vos et al., 2012; Gomez-Suaga et al., 2017). Amyotrophic lateral sclerosis (ALS)-linked protein TDP-43 disrupts MAMs by impairing the VAPB–PTPIP51 complex via activation of glycogen synthase kinase-3β (GSK3β) (Stoica et al., 2014). The fifth ER–mitochondria tethering system features PDZD8, an ER protein at MAM. PDZD8 is essential for ER-mitochondria apposition in mammalian cells by interacting with OMM protein FKBP8 (Nakamura et al., 2025). It has been shown to mediate ER to mitochondria Ca^2+^ transfer in dendrites of neurons (Hirabayashi et al., 2017). The last ER-mitochondria tethering complex involves inverted formin 2 (INF2), an ER-anchored protein. INF2 interacts with Spire1C on mitochondria to promote actin assembly at MAMs, thereby facilitating mitochondria fission (Manor et al., 2015).

**TABLE 1:** Major ER–Mitochondria tethers, functional readouts, and translational angles.

References	Tethering complex	Key functional readouts	Translational angles/therapeutic implications
Barazzuol et al. (2021), Berridge (2016), Conti et al. (2024), Szabadkai et al. (2006), Hayashi and Su (2007), Joseph et al. (1999), Myhill et al. (2008), Issop et al. (2015), Li et al. (2024a)	IP3R–GRP75–VDAC1 axis (with Sig1R, Calnexin, PACS-2, ATAD3)	Mediates ER–mitochondria Ca^2+^ transfer, supports metabolism, and regulates ROS; ATAD3 limits Ca^2+^ overload and aids cholesterol transfer	Sig1R agonists (e.g., PRE-084) enhance cell survival; GRP75/IP3R modulation prevents Ca^2+^ overload; ATAD3 targeting protects against cardiac stress
de Brito and Scorrano (2008), Munoz et al. (2013), Verfaillie et al. (2012)	Mfn2–Mfn1/Mfn2 (with PERK)	Maintains ER–mitochondria contacts and Ca^2+^ uptake; controls mitochondrial dynamics and ER stress	Mfn2 activators improve ER-mitochondrial tethering and mitophagy; PERK modulation reduces stress and apoptosis
Iwasawa et al. (2011), Namba (2019)	BAP31–FIS1–TOM40	Regulates Ca^2+^ flux and apoptosis; supports mitochondrial respiration and complex I activity	Targeting BAP31–FIS1 to modulate apoptosis and mitochondrial function
De Vos et al. (2012), Gomez-Suaga et al. (2017), Stoica et al. (2014)	VAPB–PTPIP51	Controls Ca^2+^ exchange and autophagy; disrupted in ALS via TDP-43/GSK3β pathway	Restoring VAPB–PTPIP51 or inhibiting GSK3β to protect MAM integrity in ALS
Nakamura et al. (2025), Hirabayashi et al. (2017)	PDZD8–FKBP8	Maintains ER–mitochondria proximity and neuronal Ca^2+^ signaling	PDZD8 modulation to balance neuronal Ca^2+^ handling in neurodegeneration
Manor et al. (2015)	INF2–Spire1C	Promotes actin assembly and mitochondrial fission	Actin modulators to restore fission–fusion and mitophagy balance

MAMs show tissue- and disease-specific heterogeneity, reflecting their dynamic composition and function. In aging muscle, approximately 1,300 MAM proteins display tissue- and age-specific patterns, revealing a two-phase remodeling of MAMs driven by both the tissue’s functional demands and the degenerative changes associated with aging (Lu et al., 2022). In the brains of long-term type 2 diabetic mice, proteomic analysis identified 1,313 MAM proteins, of which 144 were significantly altered, revealing diabetes-driven remodeling of the MAM proteome in this tissue (Ma et al., 2017). A gene expression–based “MAM score” in hepatocellular carcinoma effectively classified patients according to survival, metabolic, and immune characteristics. Combined with a tumor microenvironment score, it predicted genomic alterations and therapy responses, suggesting MAMs as a potential biomarker for prognosis and treatment guidance in cancer (Chen et al., 2023).

Recent advances in technologies have greatly improved our understanding of structure and composition of MAMs. Super-resolution fluorescence microscopy techniques, including stimulated emission depletion (STED) and reversible switchable optical fluorescence transition (RESOLFT) nanoscopy, have enabled the visualization of MAMs at nanometer resolution in live neuronal cells (Damenti et al., 2021). Qin et al. employed multicolor structured illumination microscopy (SIM) to visualize MAMs and mitochondrial dynamic tubules and quantify the active transport of mitochondrial DNA nucleoids across MAMs (Qin et al., 2020). Chao et al. applied split-TurboID proximity labeling to map proteins specifically enriched at MAMs with high spatial precision (Cho et al., 2020). These emerging methodologies are transforming our understanding of the molecular organization and disease relevance of MAMs.

## 3 Mechanisms of MAM-Mediated inflammation

### 3.1 Mitochondrial Ca^2+^ dysregulation and MAMs-associated inflammation

MAMs serve as essential sites in mediating Ca^2+^ transfer from ER to mitochondria. Overload or underload of Ca^2+^ within mitochondria compromises their function, leading to increased ROS generation, reduced ATP production, and the opening of the mitochondrial permeability transition pore (mPTP), ultimately triggering cell apoptosis. The consequent rise in ROS levels and apoptotic cell death induces inflammation and tissue damage (Mittal et al., 2014; Winn and Harlan, 2005). Oxidized low-density lipoprotein (ox-LDL) promoted the localization of phosphofurin acidic cluster sorting protein 2 (PACS2) at MAMs and mitochondrial Ca^2+^ overload. Silencing PACS2 blocked formation of MAMs and alleviated ox-LDL-induced apoptosis in human umbilical vein endothelial cells (Y et al., 2019). Paillard et al. reported that cyclophilin D (CypD) interacted with the VDAC1/GRP75/IP3R1 complex in cardiomyocytes, playing a regulatory role in the transfer of Ca^2+^ from ER-mitochondria. Inhibition of CypD effectively reduced mitochondrial Ca^2+^ overload and protected cardiomyocytes from hypoxia-reoxygenation-induced injury and cell death. Furthermore, disruption of ER-mitochondrial tethering via Mfn2 downregulation reduced the interaction between CypD and the IP3R1 complex, offering additional protection (Paillard et al., 2013). CypD is also a crucial regulator of the mitochondrial permeability transition pore (mPTP) opening, thereby modulating Ca^2+^-induced cell death and inflammation (Murphy, 2022). In parallel, GRP75 played a critical role in post-myocardial infarction Ca^2+^ homeostasis by enhancing ER-mitochondria coupling through the IP3R1-GRP75-VDAC1 complex. GRP75 knockdown reduced Ca^2+^ overload, MAM formation, and apoptosis in cardiomyocytes. GRP75 might be a potential therapeutic target for improving cardiomyocyte survival and inflammation after ischemic injury (Zhang et al., 2024).

Many chemicals can exploit MAM-mediated Ca^2+^ signaling to regulate inflammatory cascades. Zinc ion (Zn^2+^) has been shown to reduce cardiomyocyte apoptosis induced by ischemia-reperfusion by alleviating Ca^2+^ overload via inhibition of the IP3R1-GRP75-VDAC1 and IP3R2/FUNDC1 pathways. Silencing Bap31, VDAC1, FUNDC1, or MCU further enhanced the protective effects of zinc iron, suggesting its therapeutic potential in mitigating myocardial damage and inflammation (Guo et al., 2025). Similarly, acetylcholine has been found to alleviate hypoxia/reoxygenation-induced Ca^2+^ overload and apoptosis in endothelial cells, both of which contribute to inflammatory pathologies. This protective effect was mediated by suppression of MAM formation via disruption of the VDAC1/GRP75/IP3R1 complex (He et al., 2015). Melatonin mitigated mechanical trauma-induced liver injury by inhibiting IP3R1-dependent MAM formation via the ERK1/2-FoxO1 pathway. This action reduced mitochondrial Ca^2+^ overload, restored mitochondrial function, and alleviated apoptosis. In addition, melatonin treatment reversed trauma-induced IP3R1 upregulation, MAM formation, and cellular dysfunction *in vivo* and *in vitro* (Shi et al., 2024). Exposure of vanadium disrupted Ca^2+^ homeostasis, leading to mitochondrial Ca^2+^ overload and abnormality in MAM structure in hepatocytes. This disruption activated the NLR family pyrin domain containing 3 (NLRP3) inflammasome pathway, resulting in elevated pro-inflammatory cytokines (IL-1β, IL-18) and oxidative stress. Blocking IP3R-mediated Ca^2+^ release with 2-APB mitigated MAMs disruption and inflammation, underscoring the critical role of Ca^2+^ regulation in vanadium-induced hepatocyte injury (Zhang Y. et al., 2023). These findings emphasize that Ca^2+^ transfer through MAMs plays a pivotal role in modulating the body’s inflammatory response.

### 3.2 NLRP3 inflammasome and MAMs-associated inflammation

Building upon the role of MAM-mediated Ca^2+^ signaling in inflammation described above, emerging studies indicate that alterations in MAM integrity and function can influence NLRP3 inflammasome activation. The NLRP3 inflammasome is a multifaced innate immune sensor that recognizes foreign pathogens, cellular damage, and stress. The inflammasome complex is composed of NLRP3, pro-caspase-1, and apoptosis-associated speck-like protein (ASC), an adaptor for NLRP3 (Missiroli et al., 2018). NLRP3 forms oligomers and binds to ASC, which recruits and activates pro-caspase-1, leading to its maturation into caspase-1 and the subsequent generation of pro-inflammatory cytokines IL-1β and IL-18 (Swanson et al., 2019). The canonical NLRP3 inflammasome activation is primed by signals like toll-like receptor (TLR) ligands, resulting in elevated expression of the inflammasome components. Then, it is activated by stimuli such as viral RNA, prompting the assembly of inflammasome complex (Malik and Kanneganti, 2017). Over the past decade, MAMs have been recognized as a signaling hub for NLRP3 inflammasome activation. At the basal state, NLRP3 mainly resides in the cytosol and ER. In response to inflammasome activators such as damaged mitochondria DNA, ATP, cardiolipin, cytochrome C, and ROS, NLRP3 inflammasome is recruited to mitochondria and MAMs (Zhou et al., 2011). In THP-1 monocytes as well as BV2 microglial cells, tunicamycin-induced ER stress upregulated Mfn2 expression, increased ER–mitochondria contact sites, and promoted Ca^2+^-dependent activation of the NLRP3 inflammasome, resulting in the secretion of IL-1β (Pereira et al., 2022). After priming of J774A.1 cells with LPS, both NLRP3 and caspase-1 bound to cardiolipin, a mitochondrial lipid found on the OMM, in response to ROS. Concurrently, ASC was recruited to mitochondria and associated with NLRP3 via a Ca^2+^-dependent manner, culminating in inflammasome activation and inflammatory response (Elliott et al., 2018). Promyelocytic leukemia protein (PML), a key protein located at MAMs, formed a complex with NRLP3 and P2X7R, counteracting NLRP3 activation induced by P2X7R. Deficiency of PML intensified NLRP3-mediated inflammation and fostered tumor growth through the increased secretion of IL-1β (Missiroli et al., 2023). Sharma et al. reported that ORM1 (yeast)–like protein 3 (ORMDL3) accumulated in MAMs and elevated the formation of MAMs during inflammatory responses. This accumulation facilitated the accrual of the NLRP3 inflammasome at MAMs and enhanced IL-1β secretion. ORMDL3 also interacted with Fis1, a MAMs-associated protein, and increased mitochondrial fission. In addition, decreased expression of ORMDL3 in a colitis mouse model diminished inflammation and disease severity (Sharma et al., 2024). Mitochondrial antiviral signaling protein (MAVS), a vital MAMs-associated protein and a key player in the innate immune response, was shown to recruit NLRP3 to mitochondria during viral infection and promoted inflammasome activation (Subramanian et al., 2013). In fatty liver ischemia-reperfusion injury, ER stress triggered mitochondrial Ca^2+^ overload in macrophages through MAMs, resulting in ROS-dependent NLRP3 inflammasome activation and inflammation. Therapeutic targeting of ER stress with tauroursodeoxycholic acid (TUDCA) or mitochondrial Ca^2+^/ROS (e.g., Xestospongin-C, mito-TEMPO) inhibited NLRP3 activation (Li F. et al., 2024). Homocysteine induced NLRP3 inflammasome-dependent pyroptosis in macrophages by disrupting mitochondrial function (excessive ROS, loss of membrane potential, and diminished ATP synthesis), while simultaneously provoking ER stress. Homocysteine also elevated ER-mitochondria contacts, resulting in Ca^2+^ overload. Macrophage pyroptosis was alleviated by inhibitors for ER stress, Ca^2+^ chelation, and IP3R (Zhang S. et al., 2023).

## 4 MAMs in metabolic, neurological, and antiviral inflammation

### 4.1 MAMs in metabolic inflammation

Obesity is regarded as low-grade chronic inflammation, which plays a significant role in the onset of multiple metabolic diseases, including type 2 diabetes and NAFLD (Hildebrandt et al., 2023). The relationship between MAMs and insulin signaling remains paradoxical in the literature. While some studies suggest that increased MAM formation exacerbates insulin resistance, others demonstrate that enhancing MAM connectivity improves metabolic function. Arruda et al. reported that obesity caused a major remodeling of MAMs in the liver, leading to mitochondrial Ca^2+^ overload and oxidative stress-both critical drivers of inflammation. Additionally, downregulation of key proteins involved in ER-mitochondria interactions, such as PACS-2 and IP3R1, promoted mitochondrial function and insulin sensitivity in mice with obesity (Arruda et al., 2014). Rieussett et al. found that pharmacological inhibition of CypD disrupted the Ca^2+^ transfer between the ER and mitochondria, leading to ER stress and insulin resistance in hepatocytes. The knockout of CypD triggered the activation of PKCε, promoting lipid accumulation and development of insulin resistance in the liver (Rieusset et al., 2016). Sebastian et al. revealed that Mfn2 played a crucial role in coordinating mitochondrial and ER functions and regulating insulin signaling in liver and muscle. Liver-specific knockout of Mfn2 in mice led to ER stress, elevation of ROS, and loss of insulin sensitivity (Sebastian et al., 2012). Zhao et al. showed that oocytes from obese mice had elevated levels of MAMs and MAM-related proteins, resulting in increased mitochondrial Ca^2+^ accumulation, enhanced apoptosis, and impaired cytoplasmic maturation. Downregulation of PACS-2 reduced MAM abundance, mitochondrial Ca^2+^ levels, and apoptosis, thereby improving oocyte maturation in obese mice (Zhao et al., 2017). Zhou et al. demonstrated that connexin 43 (Cx43) bound with MCU, enhancing MCU-mediated mitochondrial Ca^2+^ uptake. Macrophage-specific knockout of Cx43 mitigated obesity by reducing inflammation in adipose tissue and limiting M1 macrophage infiltration. In addition, inhibition of Cx43 diminished the formation of MAMs and alleviated mitochondrial Ca^2+^ overload (Zhou et al., 2023). Thoudam et al. reported that pyruvate dehydrogenase kinase-4 (PDK-4) promoted the formation of MAMs, leading to mitochondrial Ca^2+^ overload and impaired insulin signaling. Conversely, reduced MAM formation in PDK4 knockout mice protected mice from insulin resistance in skeletal muscle (Thoudam et al., 2019). Tubbs et al. observed decreased interactions between IP3R1 and VDAC1, as well as between GRP75 and IP3R1, in the hepatic tissue of obese and diabetic mice. Enhancing formation of MAMs alleviated hepatic insulin resistance (Tubbs et al., 2014). Beaulant et al. reported that reduced VDAC1-IP3R1 interaction and Ca^2+^ exchange preceded impaired insulin signaling and fatty liver in obese mice, while reinforcing MAMs alleviated glucose intolerance. Additionally, hepatic VDAC1-IP3R1 interactions were reduced in obese patients with type 2 diabetes (Beaulant et al., 2022). The apparent contradiction regarding whether increased formation of MAMs promotes insulin resistance or improves insulin sensitivity can be reconciled by the notion that both inadequate and excessive ER-mitochondria tethering impair metabolic equilibrium and contribute to inflammation.

The involvement of MAMs in metabolic inflammation is further substantiated by findings in NAFLD research. Mice with liver-specific knockout of IP3R1 were protected from fatty liver development, exhibiting lower levels of mitochondrial Ca^2+^, hepatic triglycerides, and lipid droplet formation. In patients with NAFLD, elevated IP3R1 expression and increased ER-mitochondrial colocalization were observed in the liver (Feriod et al., 2017). On the other hand, reduced IP3R2 expression was observed in fat-loaded Huh7 cells, liver tissue from NAFLD rat models, and liver biopsies from patients with NAFLD. Liver regeneration was impaired in NAFLD through a c-Jun-mediated decrease in IP3R2, which was crucial for Ca^2+^ signaling in hepatocytes (Khamphaya et al., 2018). Interestingly, Mfn2 was shown to play a protective role in liver disease, as decreased levels of Mfn2 were shown in liver biopsies from patients with non-alcoholic steatohepatitis (NASH) and in mouse models of the condition. The absence of Mfn2 disrupted the transfer of phosphatidylserine (PS) to mitochondria, impairing phospholipid synthesis and inducing ER stress, ultimately contributing to the development of NASH (Hernandez-Alvarez et al., 2019). Hepatic stimulator substance (HSS) protected the liver from NASH by maintaining the activity of sarco/ER Ca^2+^ ATPase (SERCA) in the MAMs, preventing the excessive flow of cytosolic free Ca^2+^ into the mitochondria. This protective mechanism preserved mitochondrial function and alleviated palmitic acid-induced hepatocyte steatosis (Xiao et al., 2017). Nanoplastic exposure promoted the progression from simple steatosis to NASH by upregulating Fatp2, which enhanced the assembly of MAMs via binding with IP3R1, leading to mitochondrial Ca^2+^ overload and oxidative stress (Wei et al., 2024). CDP-DAG synthase 2 (CDS2) has been identified at MAMs, where it maintains integrity and function of MAMs. CDS2 deficiency disrupted MAM protein composition and reduced mitochondrial phosphatidylethanolamine (PE) levels, leading to impaired mitochondrial function. This metabolic disturbance promoted the development of hepatic steatosis, inflammation, and fibrosis, thereby driving NASH progression (Xu et al., 2022). Quantitative analysis of MAMs in NAFLD patient hepatocytes, via IP3R1-VDAC1 proximity ligation assay (PLA), revealed a significant correlation with key histological features, including hepatocyte ballooning, inflammation, and fibrosis. MAM abundance may serve as a discriminative marker, differentiating NAFLD from NASH, underscoring its potential for disease stratification (Jin et al., 2022).

### 4.2 MAMs in neurological inflammation

Parkinson’s disease (PD) arises from a complex interplay of factors, including chronic neuroinflammation, mitochondrial dysfunction, and impaired proteostasis, which collectively contribute to neurodegeneration and the progressive depletion of dopamine neurons within the substantia nigra (Arena et al., 2022). α-synuclein (α-Syn) is abundantly produced in neurons to regulate synaptic function. However, its overexpression or the presence of disease-associated mutations disrupts its normal function, leading to toxic aggregates that form Lewy bodies, a pathological feature associated with Parkinson’s disease (Barbuti, 2024). A mouse model with overexpression of α-Syn exhibited progressive degeneration of dopaminergic neurons, accompanied by microglial phagocytic exhaustion and excessive production of oxidative and proinflammatory cytokines (Bido et al., 2021). Although α-Syn is predominantly localized in the cytosol, emerging evidence reveals its presence in MAMs. Pathogenic α-Syn point mutations impair its interaction with MAMs, leading to impaired ER-mitochondria apposition and diminished MAM function (Guardia-Laguarta et al., 2014). Wild-type α-Syn impairs mitochondrial recovery following MPP + neurotoxin-induced stress and suppresses mitochondrial Ca^2+^ uptake (Ramezani et al., 2023). α-Syn interacts with VAPB, and its overexpression or Parkinson’s-associated mutations disrupts the VAPB–PTPIP51 interaction, thereby diminishing the contact sites between ER and mitochondria. Consequently, Ca^2+^ transfer between the two organelles is impaired, resulting in reduced mitochondrial ATP production (Paillusson et al., 2017). In addition, α-Syn dynamically associates with VDAC1, altering its channel conductance to Ca^2+^ permeability (Rosencrans et al., 2021). Aggregated α-Syn has been shown to interact with MAM proteins calnexin and GRP94 in dopaminergic neurons, resulting in ER stress, a key initiator of inflammatory response (Stojkovska et al., 2022). Accumulation of α-Syn induces ER stress and activates the unfolded protein response in the neurons, primarily through PERK, ATF6, and CHOP signaling pathways, thereby triggering a proinflammatory response. Nevertheless, overexpression of MAM protein GRP78 mitigates α-syn-induced neurotoxicity by forming a protective complex with α-syn and reducing neuronal apoptosis (Gorbatyuk et al., 2012). Other MAM proteins might also participate Parkinson’s disease pathogenesis. For instance, knockout of PARK2, which encodes the E3 ligase Parkin, elevated ER–mitochondria proximity in fibroblasts. Patient-specific induced pluripotent stem cells-derived neurons harboring PARK2 mutations exhibited markedly increased Ca^2+^ flux from the ER to mitochondria (Gautier et al., 2016).

There are two hallmark pathologies to define Alzheimer’s disease: the aggregates of hyperphosphorylated Tau protein within neurons and the deposits of β-amyloid (Aβ) outside cells, both arising from disrupted protein processing mechanisms (Zheng and Wang, 2025). However, growing evidence highlights neuroinflammation as a key contributor to its development (Heneka et al., 2024). ApoE4, which primarily functions in lipid transport, is a key genetic risk factor for Alzheimer’s disease. Compared to ApoE3, ApoE4 significantly increases MAM activity in cells treated with conditioned media containing ApoE3 or ApoE4, suggesting that ApoE4 enhances ER–mitochondrial communication (Tambini et al., 2016). ApoE interacts with MAM proteins such as TOMM40, LONP1, and VDAC1, indicating its potential role in ER–mitochondrial connectivity and function (Rueter et al., 2024). Presenilins 1 and 2 (PS1 and PS2) form the enzymatic center of the γ-secretase complex, which cleaves amyloid precursor protein (APP) to generate Aβ peptides (De Strooper et al., 2012). PS1 as well as PS2 are widely distributed inside the cells and particularly concentrated in MAMs (Area-Gomez et al., 2009). Familial Alzheimer’s disease (FAD)-associated mutants of PS2, not PS1, enhance the movement of Ca^2+^ between the ER and mitochondria by strengthening the physical coupling between these two organelles in neurons (Zampese et al., 2011). FAD-associated PS2 mutants exhibit elevated binding for Mfn2, leading to stronger ER–mitochondria tethering and more efficient Ca^2+^ transfer to mitochondria compared to the wild-type protein (Filadi et al., 2016). This elevated Ca^2+^ transfer exacerbates mitochondrial dysfunction and oxidative stress, key triggers of neuroinflammatory pathways (Picca et al., 2020). Additionally, both APP and Aβ localize to MAMs, where they undergo processing by active β- and γ-secretases. Overexpression of FAD-linked mutant APP boosts ER–mitochondria contacts and leads to increased lipid accumulation (Del Prete et al., 2017). MAMs are believed to act as detergent-resistant lipid rafts, offering a dynamic and stable platform that facilitates the activity of both β- and γ-secretases (Area-Gomez et al., 2012). In JNPL3 mice with the tau P301L mutation, there was an increase in ER-mitochondria contacts within motor neurons, linked to elevated levels of phosphorylated tau specifically on ER membranes. A similar increase in phosphorylated tau at the ER was also seen in brain samples from Alzheimer’s disease patients, suggesting a common mechanism that may contribute to neurodegeneration (Perreault et al., 2009). However, another study showed that the P301L tau mutation disrupted ER-mitochondria interactions by weakening the VAPB–PTPIP51 tethering, resulting in impaired mitochondrial cholesterol metabolism and decreased pregnenolone production (Szabo et al., 2023). Accumulation of human tau protein augments mitochondrial fusion by upregulating Mfn1 and Mfn2 in rat hippocampal neurons, leading to perinuclear mitochondrial clustering, reduced ATP production, and impaired cell viability (Li et al., 2016). These Tau-induced disruptions in mitochondrial dynamics and bioenergetics promote neuronal susceptibility to inflammatory stimuli, driving glial activation and neuroinflammation in Alzheimer’s disease.

### 4.3 MAMs in antiviral inflammatory response

When viral RNA or DNA enters infected cells, they are detected by retinoic acid-inducible gene-I (RIG-1, for RNA) and cyclic GMP-AMP synthase (cGAS, for DNA) in cytosol. RIG-1 activates its adaptor protein, MAVS, which is a MAM protein (Kumar et al., 2006). Once activated, MAVS recruits members of the TNF receptor associated factor (TRAF) family, initiating a signaling cascade that leads to the phosphorylation of interferon regulatory factor 3 (IRF3) and its subsequent nuclear translocation. This process stimulates the expression of type I/III interferon (IFN) genes, along with a range of proinflammatory cytokines, which are critical for innate immune response (Bender et al., 2015). The elevated levels of proinflammatory cytokines can contribute to tissue damage and systemic inflammation. Stimulator of interferon genes (STING), a known MAM-localized protein, binds with the RIG-I and MAVS complex, with this interaction becoming more pronounced in response to infection (Ishikawa and Barber, 2008). Similarly, cytosolic DNA triggers the cGAS-STING pathway. Activated STING undergoes ER-to-Golgi trafficking and recruits TANK-binding kinase 1 (TBK1). TBK1 then phosphorylates IRF3, driving type I/III IFN production and amplifying inflammatory response (Dvorkin et al., 2024). The sustained activation of cGAS-STING-TBK1-IRF3 pathway has been implicated in self-DNA-induced lung inflammation (Benmerzoug et al., 2018).

During the infection of Japanese encephalitis virus, STING deletion impairs the activation of the IRF3/IFN signaling pathway (Nazmi et al., 2012). Gp78, a MAM protein and an E3 ubiquitin ligase, modulates MAVS-mediated antiviral signaling via two potential mechanisms. First, Gp78 degrades MAVS via ER-associated degradation (ERAD). Second, Gp78 directly associates with MAVS and exerted its protein degradation (Jacobs et al., 2014). By damping MAVS signaling, Gp78 may indirectly limit the overproduction of proinflammatory molecules, serving as a checkpoint in inflammation control. vMIA, a protein synthesized by the human cytomegalovirus (HCMV) of the Herpesviridae family, associates with the MAMs through its cholesterol-binding domain. This interaction allows vMIA to bind to the Sig1R which modulates the activity of IP3R at the MAMs, thereby influencing Ca^2+^ transfer to mitochondria (Williamson et al., 2011). Additionally, HCMV upregulates the expression of GRP75, resulting in mitochondrial Ca^2+^ accumulation and driving the induction of apoptosis (Bozidis et al., 2010). Hepatitis C virus (HCV) targets MAMs by utilizing its NS3/4A serine proteases, which cleaves MAVS to suppress the IFN-mediated antiviral response. The NS3/4A protease complex associates with intracellular membranes, including those at the MAMs, specifically targeting MAVS (Horner et al., 2012). Additionally, other HCV proteins, such as core protein, elevate mitochondrial ROS by stimulating the MCU (Li et al., 2007). The human immunodeficiency virus (HIV)-1 Tat protein disrupts the function of MAMs by inducing the phosphorylation of PTPIP51, thereby impairing its interaction with VAPB. This disruption triggers the accumulation of ROS and induces mitochondrial stress, contributing to premature brain aging in HIV patients (Arjona et al., 2023). Concurrently, the oxidative stress and mitochondrial dysfunction can further promote neuroinflammation. Additionally, the HIV-1 viral protein R (Vpr) is synthesized in the ER and trafficked to the mitochondria through MAMs, a process that is dependent on proteins including Mfn2, dynamin-related protein 1 (DRP1), and ATAD3A. Vpr downregulates Mfn2 and compromises the mitochondrial outer membrane, resulting in mitochondrial fragmentation and cell apoptosis (Huang et al., 2012). This Vpr-induced apoptosis may release damage-associated molecular patterns and proinflammatory signals, contributing to systematic inflammation (Li et al., 2020).

## 5 Role of MAMs in acute lung injury

**FIGURE 2:**
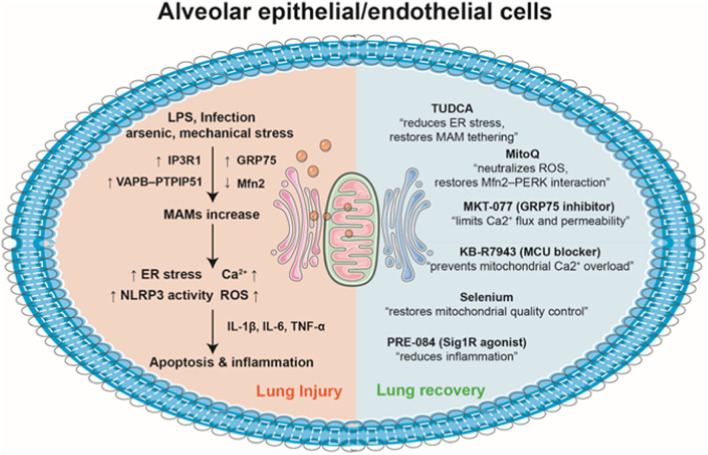
MAMs in the pathogenesis and therapeutic modulation of acute lung injury. (1) Pathological stimuli such as LPS, infection, arsenic, and mechanical stress alter MAM proteins (upregulation of IP3R1, GRP75, and the VAPB–PTPIP51 complex, and downregulation of Mfn2), leading to excessive ER–mitochondria contacts in alveolar epithelial/endothelial cells. This promotes elevated Ca^2+^ transfer via IP3R–GRP75–VDAC1 complexes, resulting ER stress, Ca^2+^ overload, NLRP3 inflammasome activation, and overproduction of ROS. Consequently, the release of proinflammatory cytokines, including IL-1β, IL-6, and TNF-α, amplifies cellular injury, culminating in apoptosis and the development of acute lung injury. (2) Pharmacological interventions such as TUDCA, MitoQ, MKT-077, KB-R7943, selenium, and PRE-084 restore balanced ER–mitochondria contacts. There agents regulate Ca^2+^ flux, preserve mitochondrial integrity, attenuate ROS production, and mitigate inflammatory signaling. By normalizing MAM tethering and supporting mitochondrial dynamics, these therapeutic strategies promote cellular homeostasis and facilitate lung tissue recovery.

In acute lung injury, perturbation of MAMs disturbs ER–mitochondrial Ca^2+^ signaling and mitochondrial function, precipitating oxidative stress, inflammation, and vascular leakage. Dong et al. showed that intravenous administration of lipopolysaccharide (LPS) resulted in acute lung injury, which was accompanied by an enhanced formation of MAMs and a decrease in the respiratory control ratio, a measure of mitochondria function. There was also an increase of IP3R1 levels in MAM and lung lysate. In MLE-12 lung epithelial cells, levels of mitochondrial Ca^2+^ and production of ROS were elevated after LPS treatment, resulting in a reduction in mitochondrial membrane potential. However, these LPS-induced alterations were partially mitigated by knockdown of IP3R1 expression in MLE-12 cells (Dong et al., 2023) (Table 2) (Figure 2). In a mouse model of silicosis, silica reduced VAPB and PTPIP51 expression and disrupted their co-localization, indicating impaired MAMs. Inhibition ER stress with TUDCA and dioscin in silica-exposed fibroblasts restored ER-mitochondria tethering, mitigated mitochondrial damage, and reduced MAM disruption. Conversely, knockdown of VAPB or PTPIP51 aggravated mitochondrial dysfunction, which was accompanied by heightened ER stress and a significant increase in fibroblast activation (Ban et al., 2025). Titanium dioxide nanoparticles evoked ER stress in human bronchial epithelial cells. These nanoparticles disrupted the function of mitochondria by reducing the expression of IP3R, VDAC1, and GRP75, leading to reduced levels of mitochondrial Ca^2+^ and ATP. Additionally, these nanoparticles promoted autophagy, as indicated by a higher LC3 II/LC3 I ratio along with elevated levels of sequestosome-1 and BECN1. However, their effects on mitochondrial function were alleviated by inhibiting ER stress with TUDCA (Yu et al., 2015). In a separate study, acute arsenic exposure was shown to elicit ferroptosis, impair lung function, and trigger acute lung injury in mice. Arsenic treatment reduced the expression of MAM-associated Mfn2 while PERK phosphorylation was elevated, leading to decreased Mfn2-PERK interaction and impaired MAM formation in lung epithelial cells and the lung. MitoQ, a neutralizer for mitochondrial ROS, promoted the interaction between Mfn2 and PERK. Furthermore, MitoQ alleviated the ferroptosis and acute lung injury caused by arsenic exposure (Li et al., 2022). GRP78, a protein localized at mitochondria-MAMs, plays a pivotal role in inflammatory responses. In a mouse model of LPS inhalation, treatment with SubAB, a specific inactivator for GRP78, significantly lowered VCAM-1 and IL-1β levels, reduced neutrophil infiltration, diminished albumin levels in the bronchoalveolar lavage (BAL), and alleviated lung tissue edema. Gene transfer of a dominant-negative GRP78 mutant into the lung endothelium conferred protection against LPS-evoked lung inflammation (Leonard et al., 2019).

**TABLE 2:** Studies demonstrating the roles of MAMs in acute lung injury in animal models.

References	Animal model	MAM proteins	Major findings	Mechanisms
Dong et al. (2023)	LPS-induced lung injury	IP3R1	↑Formation of MAMs in the lung tissue ↑IP3R1 levels in MAM and lung lysate ↑ Mitochondrial Ca^2+^ and production of ROS, as well as ↓mitochondrial membrane potential *in vitro*.	IP3R1 mediates LPS-induced MAMs formation and mitochondrial malfunction.
Ban et al. (2025)	Mouse model of silicosis	VAPB-PTPIP51	↓VAPB and PTPIP51 level and colocalization in lung tissue ↓Mitochondrial damage and MAM disruption by TUDCA and dioscin in silica-exposed fibroblasts.	SiO_2_ disrupts VAPB-PTPIP51 tethering during progression of lung injury and fibrosis.
Li et al. (2022)	Arsenic-induced acute lung injury	Mfn2 and PERK	↑PERK phosphorylation and ↓Expression of Mfn2 ↓Mfn2-PERK interaction and MAM formation ↓Ferroptosis and arsenic-induced lung injury with MitoQ treatment	MAMs dysfunction is responsible for arsenic-induced ferroptosis and acute lung injury.
Leonard et al. (2019)	LPS-induced acute lung injury	GRP78	↓VCAM-1, IL-1β, neutrophil infiltration, and albumin levels in BAL by a GRP78 inactivator ↓Lung inflammation by gene transfer of a dominant-negative GRP78 mutant to lung endothelium.	Inactivation of MAM-associated protein GRP78 alleviates acute lung injury via decreasing endothelial inflammation.
Rimessi et al. (2020)	Lung infection in cystic fibrosis	VAPB-PTPIP51	↑VAPB-PTPIP51 tethering, mitochondrial Ca^2+^ uptake, and NLRP3 activation in bronchial cells from cystic fibrosis patients ↑survival and ↓bacterial burden in mice with CFTR knockout after MCU inhibition	Lung infection in cystic fibrosis elevates VAPB-PTPIP51 tethering, amplifying inflammation.
Mahamed et al. (2023)	Mouse model of CLP-induced lung injury	Sig1R	↑Inflammatory response in lung endothelial cells by Sig1R knockdown *in vitro* ↓CLP-induced lung injury with reduced neutrophil infiltration and lung edema by Sig1R agonist	MAM-associated Sig1R alleviates acute lung injury by reducing endothelial inflammation.
Ye et al. (2021)	Mouse model of ventilator-induced Lung Injury	IP3R	↑IP3R levels in MAMs and elevated MAM formation ↑Lung injury by IP3R agonist ↓Lung injury by IP3R antagonist	Both IP3R inhibitor and Ca^2+^ chelator alleviates ventilator-induced lung injury
Leonard et al. (2020)	Mouse model of LPS-induced acute lung injury	GRP75	↓Lung injury by GRP75 inhibitor MKT-077 ↓Ca^2+^ signaling and endothelial cell permeability by GRP75 inhibition/depletion *in vitro*	GRP75 promotes endothelial cell dysfunction in acute lung injury
Yang et al. (2022), Carpio et al. (2021)	SARS-CoV-2 M protein-induced lung injury in mice	BOK and IP3R	↑BOK level and apoptosis in lung epithelial cells ↑Alveolar-capillary permeability and lung injury ↑Transfer of Ca^2+^ from the ER to mitochondria after BOK interact with IP3R	Membrane protein of SARS-CoV-2 triggered mitochondrial apoptosis and lung injury via BOK.
Pan et al. (2021), Wu et al. (2016)	LPS-induced acute lung injury	FUNDC1	↑Lung FUNDC1 level and inflammatory response. ↑NLRP3 activation and lung injury in FUNDC1 knockout mice.	Knockout of MAM-associated FUNDC1 potentiates inflammasome-mediated lung injury.
Lin et al. (2023)	Cadmium-induced lung injury in sheep	FUNDC1	↑Lung injury, mitochondrial dysfunction, oxidative stress, and FUNDC1-mediated mitophagy. ↓Lung injury by selenium treatment.	Selenium alleviates cadmium-induced lung injury by modulating mitochondrial quality control via FUNDC1.

TUDCA, tauroursodeoxycholic acid; BAL, bronchoalveolar lavage; NLRP3, NOD-, LRR- and, pyrin domain-containing protein 3; MCU, mitochondrial Ca^2+^ uniporter; CFTR, cystic fibrosis transmembrane conductance regulator; CLP, cecal ligation and puncture; BOK, BCL-2, ovarian killer.

In cystic fibrosis, *P. aeruginosa* infection in bronchial cells from cystic fibrosis patients enhanced VAPB-PTPIP51 tethering at MAMs, disrupting autophagy and increasing mitochondrial Ca^2+^ uptake, which triggers NLRP3 inflammasome activation and hyperinflammation. Inhibition of the MCU with KB-R7943 restored autophagy and reduced inflammation *in vitro*. KB-R7943 treatment improved survival and reduced *P. aeruginosa* bacterial burden in mice with cystic fibrosis transmembrane conductance regulator (CFTR) knockout, demonstrating efficacy in cystic fibrosis-specific lung infection (Rimessi et al., 2020). Knockdown of Sig1R, a MAM protein and binding partner of GRP78, exacerbated the LPS-induced proinflammatory responses, with upregulation of ICAM-1, VCAM-1, and IL-8 in primary pulmonary endothelial cells. Conversely, the Sig1R agonist PRE-084 attenuated this inflammatory response by dissociation Sig1R from GRP78. In a mouse model of cecal ligation and puncture (CLP)-induced lung injury, intraperitoneal administration of PRE-084 reduced levels of ICAM-1 and IL-6 in the lung homogenate, decreased neutrophil infiltration, and alleviated lung edema (Mahamed et al., 2023). Mouse lungs exposed to mechanical ventilation with high tidal volume displayed higher IP3R levels in MAMs and elevated MAM formation, compared with spontaneous breathing. This high tidal volume also disturbed intracellular Ca^2+^ homeostasis, leading to heightened Ca^2+^ flux from ER to mitochondria. Treatment with carbachol, an IP3R agonist, exacerbated lung injury induced by high tidal volume. Nevertheless, pretreatment with 2-APB, an IP3R inhibitor, or BAPTA-AM, a Ca^2+^ chelator, significantly mitigated lung injury (Ye et al., 2021). In an aerosolized LPS model of acute lung injury, GRP75 inhibitor MKT-077 alleviated neutrophil infiltration, minimized microvascular leakage, and reduced the expression of IL-1β, E-selectin, and TNFα. In addition, GRP75 inhibition/depletion blocked thrombin-mediated Ca^2+^ signaling and endothelial cell permeability *in vitro* (Leonard et al., 2020). By blocking ubiquitination, SARS-CoV-2 membrane protein promoted the cellular accumulation of BCL-2 ovarian killer (BOK), a MAM protein. This resulted in BOK-driven mitochondrial apoptosis in lung epithelial cells, contributing to heightened alveolar-capillary permeability and lung injury in mice (Yang et al., 2022). Mechanistically, BOK is situated in the ER and participates in Ca^2+^ signaling via interaction with IP3R. BOK deficiency decreased ER-mitochondrial contact sites, hindered Ca^2+^ transfer, and diminished bortezomib-induced apoptosis (Carpio et al., 2021). In the lung of LPS-treated mice, levels of FUNDC1, a MAM protein, and inflammatory response/apoptosis were elevated compared to normal controls. FUNDC1 deficiency promoted NLRP3 inflammasome activation (NLRP3, caspase-1, IL-1β, ASC) and exacerbated LPS-induced lung injury relative to wild-type mice (Pan et al., 2021). FUNDC1 was abundantly present in MAMs via binding with ER protein calnexin and participated in various cellular processes, including autophagy, mitophagy, and mitochondria fission (Wu et al., 2016). Cadmium induced lung injury in sheep by triggering mitochondrial dysfunction, oxidative stress, and FUNDC1-mediated mitophagy. Selenium supplementation counteracted these effects by restoring mitochondrial quality control, reducing oxidative stress, and inhibiting excessive mitophagy, thereby alleviating cadmium-induced lung injury (Lin et al., 2023).

A central unresolved question in the field concerns whether MAM dysfunction serves as a primary driver of lung injury or emerges as a downstream consequence of cellular stress. Evidence from diverse experimental models suggests that both mechanisms are plausible and context dependent. For instance, loss of Mfn2 at MAMs disrupts phosphatidylserine transfer, leading to a NASH-like phenotype and liver cancer, thereby demonstrating a causal role for MAM dysfunction in disease pathogenesis (Hernandez-Alvarez et al., 2019). Similarly, either loss or overexpression of GRP75 or Mfn2 in hepatocytes results in triglyceride accumulation, further highlighting the direct impact of MAM imbalance on cellular metabolism and organ pathology (Bassot et al., 2021). Conversely, under conditions of ER stress, upregulation of the IP3R1–GRP75–VDAC1 complex enhances ER–mitochondrial Ca^2+^ transfer and promotes mitochondrial oxidative injury, indicating that MAM alterations may also arise secondarily to cellular stress (Yuan et al., 2022). Collectively, these findings underscore the bidirectional relationship between MAM integrity and cellular homeostasis, suggesting that MAM dysfunction can act both as a precipitating factor and as a pathological consequence in acute lung injury and other diseases.

## 6 MAMs as emerging therapeutic targets in acute lung injury

Although a growing body of literature has implicated MAM dysfunction in the pathogenesis of acute lung injury, it remains unclear whether current clinical therapies directly influence MAM integrity or signaling. Existing treatments for ARDS—such as lung-protective mechanical ventilation, tailored use of corticosteroids, and conservative fluid management—are primarily supportive and do not specifically target MAM pathways. To date, no approved therapy has been developed with the explicit aim of modulating MAM structure or function. However, several experimental or repurposed agents have been shown to indirectly influence MAM-associated processes by acting on mitochondria, ER, or MAMs (Figure 2). For instance, TUDCA, an ER stress inhibitor, restores ER–mitochondria tethering and normalizes mitochondrial function in silica- and titanium dioxide nanoparticle–induced lung injury models (Ban et al., 2025) (Yu et al., 2015). MitoQ, a mitochondria-targeted antioxidant, enhances Mfn2–PERK interaction and maintains MAM integrity in arsenic-induced lung injury (Li et al., 2022). Similarly, PRE-084, a Sig1R agonists, alleviatea sepsis-induced lung injury through dissociation of Sig1R from GRP78, thereby modulating MAM-associated proinflammatory signaling (Mahamed et al., 2023). KB-R7943, a MCU blocker, enhances survival rate and decreases *P. aeruginosa* load in cystic fibrosis by blocking MAM hyperactivation and restoring autophagy (Rimessi et al., 2020). Moreover, MKT-077, a GRP75 inhibitor, attenuates LPS-induced lung injury via suppression of excessive ER–mitochondrial Ca^2+^ transfer (Leonard et al., 2020). Collectively, while no current therapies specifically and directly target MAM-related pathways, the emerging mitochondrial-, ER-, and MAM-focused interventions may provide new opportunities for developing MAM-targeted therapies in acute lung injury.

## 7 Conclusion

MAMs have emerged as critical regulatory hubs that integrate signals between the ER and mitochondria. Evidence from metabolic, neurodegenerative, and antiviral disease models supports a broader role for MAMs in modulating inflammation across diverse pathophysiological settings. In acute lung injury, dysregulated ER–mitochondria communication at MAMs precipitates mitochondrial Ca^2+^ overload, intensifies oxidative stress, and amplifies inflammatory signaling—processes that collectively drive endothelial barrier disruption and worsen pulmonary inflammation. Future research aimed at elucidating the precise molecular architecture of MAMs and their dynamic regulation during stress and inflammation will be essential for translating these insights into clinical therapies. Overall, MAMs represent a novel and compelling target for innovative strategies to mitigate the impact of acute lung injury and potentially other inflammation-driven diseases.
